# 

**Published:** 1897-03

**Authors:** 


### CONTENTS:

### SELECTIONS.

Article I.—A Plea for Conservative Oral Surgery with Practical Illustrations. By G. Lenox Curtis. - - 481

" II.—Cataphoresis. By Dr. Cyrus A. Allen. - - 494

" III.—Pyorrhea Alveolaris From a New Standpoint. By William H. Trueman, D. D. S. - - - - 502

" IV.—Edison Current in Cataphoresis. By L. E. Custer, B. S., D. D. S. - - - - - - - 513

### EDITORIAL, ETC.

A Criticism of the Editorial "Dental Faculties in Medical Schools." 517

Committees for Tri-union Meeting of the Washington City Dental Society, Maryland State Dental Association and the Virginia State Dental Association, 1897. - 520

### MONTHLY SUMMARY.

Diagnosis of Pulpititis. - - - - - - - 523

Nitrate of Silver. - - - - - - - - - 524

Pulpitis vs. Peridontitis. - - - - - - - 526

Nuclein in Dental Practice. - - - - - - 526

Pulpitis. - - - - - - - - - 527

Chloroform. - - - - - - - - - 527

"Cataphoresis." - - - - - - - - 527

"Murder (and Other Crimes) Will Out." - - - - 528

A Good Prescription. - - - - - - - 528

Whooping Cough Bacillus. - - - - - - 528

An Indian Has a Tooth Filled. - - - - - - 528

